# Are *APOE* ɛ genotype and *TOMM40* poly-T repeat length associations with cognitive ageing mediated by brain white matter tract integrity?

**DOI:** 10.1038/tp.2014.89

**Published:** 2014-09-23

**Authors:** D M Lyall, S E Harris, M E Bastin, S Muñoz Maniega, C Murray, M W Lutz, A M Saunders, A D Roses, M del C Valdés Hernández, N A Royle, J M Starr, D J Porteous, J M Wardlaw, I J Deary

**Affiliations:** 1 Centre for Cognitive Ageing and Cognitive Epidemiology (CCACE), University of Edinburgh, Edinburgh, UK; 2 Brain Research Imaging Centre, Division of Neuroimaging Sciences, University of Edinburgh, Edinburgh, UK; 3 Department of Psychology, University of Edinburgh, Edinburgh, UK; 4 Medical Genetics Section, University of Edinburgh Centre for Genomics and Experimental Medicine and MRC Institute of Genetics and Molecular Medicine, Western General Hospital, Crewe Road, Edinburgh, UK; 5 Department of Neuroimaging Sciences, Scottish Imaging Network, A Platform for Scientific Excellence (SINAPSE) Collaboration, The University of Edinburgh, Edinburgh, UK; 6 Joseph & Kathleen Bryan Alzheimer's Disease Research Center, Department of Neurology, Duke University Medical Center, Durham, NC, USA; 7 Zinfandel Pharmaceuticals, Durham, NC, USA; 8 Alzheimer Scotland Dementia Research Centre, Edinburgh, UK

## Abstract

Genetic polymorphisms in the *APOE* ɛ and *TOMM40* ‘523' poly-T repeat gene loci have been associated with significantly increased risk of Alzheimer's disease. This study investigated the independent effects of these polymorphisms on human cognitive ageing, and the extent to which nominally significant associations with cognitive ageing were mediated by previously reported genetic associations with brain white matter tract integrity in this sample. Most participants in the Lothian Birth Cohort 1936 completed a reasoning-type intelligence test at age 11 years, and detailed cognitive/physical assessments and structural diffusion tensor brain magnetic resonance imaging at a mean age of 72.70 years (s.d.=0.74). Participants were genotyped for *APOE* ɛ2/ɛ3/ɛ4 status and *TOMM40* 523 poly-T repeat length. Data were available from 758–814 subjects for cognitive analysis, and 522–543 for mediation analysis with brain imaging data. *APOE* genotype was significantly associated with performance on several different tests of cognitive ability, including general factors of intelligence, information processing speed and memory (raw *P*-values all<0.05), independently of childhood IQ and vascular disease history. Formal tests of mediation showed that several significant *APOE*-cognitive ageing associations—particularly those related to tests of information processing speed—were partially mediated by white matter tract integrity. *TOMM40* 523 genotype was not associated with cognitive ageing. A range of brain phenotypes are likely to form the anatomical basis for significant associations between *APOE* genotype and cognitive ageing, including white matter tract microstructural integrity.

## Introduction

Alzheimer's disease (AD) is a progressive neurodegenerative disease characterized by cognitive impairment. Two genetic risk factors for late onset Alzheimer's disease are in the apolipoprotein-e (*APOE*; http://www.ncbi.nlm.nih.gov/gene/348) and translocase of outer mitochondrial membrane 40 (*TOMM40)* gene poly-T repeat loci (http://www.ncbi.nlm.nih.gov/gene/10452).^1,2^

Previous studies have investigated associations between *APOE* genotype and cognitive ability in non-demented older adults. Wisdom *et al.*^3^ conducted a meta-analysis of 77 studies, excluding samples with any disorders that may affect cognitive ability such as dementia or Parkinson's disease (final *N*=40 942; weighted estimate mean age=63.14 years, s.d.=13.10). For analytic purposes, they then constructed a ‘d' effect size metric that weighted standardized differences between the groups in terms of sample sizes. Significant deleterious effects of the ɛ4 allele were found for cognitive domains of episodic memory, global cognitive function, executive function and perceptual speed (all *P*<0.05). There were no significant effects on verbal ability, primary memory, attention or visuospatial functioning.

The association between *APOE* ɛ4 and cognitive ability may be modified by other genetic variables that exert independent effects.^4^ The *TOMM40* 523 poly-T repeat locus has recently been significantly associated with brain-related phenotypes (for example, cognitive decline), independent of *APOE* genotype.^5^ The independent effects of this locus on older-age cognitive ability have been examined in three studies. The largest of these was by Caselli *et al.*^6^ who reported a deleterious effect of the Very-long/Very-long genotype (vs Short/Short) on the amount of longitudinal change in the auditory verbal learning test (*N*=639; genotype mean ages=57.8–60.9 years; mean duration of follow-up=6.1 years±3.1; *P*=0.04 in *APOE* ɛ3/ɛ3 subgroup). Two other smaller reports by Hayden *et al.*^7^ (*N*=127) and Johnson *et al.*^8^ (*N*=117) have also reported some specific significant effects of *TOMM40* 523 genotype on cognitive ability in older adults.

One of the main aims of this paper is to investigate brain imaging variables that might mediate previously established genetic-cognitive associations; it is important to understand the anatomical brain substrates of cognitive ageing. Penke *et al.*^9^ investigated the role of white matter integrity using different metrics obtained from diffusion tensor magnetic resonance imaging (DT-MRI), one of which was fractional anisotropy (FA; where lower FA reflects reduced brain white matter integrity). They reported that a general factor of FA constructed with principal components analysis (PCA) was significantly associated with general factors of processing speed (*g*_speed_; standardized *β*=−0.19) and general cognitive ability (*g*; standardized *β*=0.13) in the Lothian Birth Cohort 1936 (LBC1936), explaining around 10% of the variance in general cognitive ability. Specifically, lower FA scores were associated with worse general cognitive ability, and slower information processing speed.

A previous report by Lyall *et al.*^10^ tested for associations between *APOE/TOMM40* 523 genotypes and tract-averaged FA values in several brain white matter tracts in the 73-year-old LBC1936 (the same sample whose data are analysed in the present study), assessed by DT-MRI. We found significant and independent deleterious effects of the *APOE* ɛ4 and *TOMM40* 523 ‘Short' (vs Long/Very-long) alleles on specific tracts, independent of the covariates of age, gender, vascular disease history and childhood intelligence. For *APOE* genotype, these tracts were the left ventral cingulum and the left inferior longitudinal fasciculus, and for *TOMM40* 523, the left uncinate fasciculus, left rostral cingulum, left ventral cingulum and G_FA_. The statistically significant association with G_FA_—which reflects shared variance among the examined tracts—may indicate a general effect of *TOMM40* 523 on white matter tract integrity in the brain. It is unclear why tract-specific measures show significant deleterious effects of genetic variation at the *APOE/TOMM40* gene loci, compared with other white matter tracts.^10^ These specific tracts may be particularly sensitive to injury or pathology.^11^ (Note however that group differences in white matter FA may be due to differences in, for example, axon diameter, packing density or membrane permeability, and in this sense lower FA may not necessarily reflect lower microstructural integrity *per se*.^12^ ) In that report (Lyall *et al.*^10^), we did not assess the effects of *APOE/TOMM40* 523 genotypes on cognitive ability, and we are aware of no studies that formally test the extent to which deleterious effects of *APOE* ɛ4 on cognitive ageing are mediated by brain white matter tract integrity (although see Ryan *et al.*^13^).

The present study therefore aims to: (1) assess the effects of *APOE/TOMM40* 523 genotypes on cognitive abilities in older people, at first unadjusted and then adjusted for age 11 intelligence (that is, reflecting cognitive ageing); and (2) determine the extent to which any such significant associations are mediated by previously reported associations between these gene loci and white matter tract integrity (FA), assessed with DT-MRI in this same sample.^10^

## Materials and Methods

### Sample

The LBC1936 is a longitudinal ageing sample of generally healthy, community-dwelling older adults.^14,15^ Briefly, most of the LBC1936 sample completed the Moray House Test (no.12) of verbal reasoning as part of the Scottish Mental Survey 1947 at a mean age of 11 years, and were recruited for detailed cognitive, medical and demographic assessments at the Wellcome Trust Clinical Research Facility (WTCRF, Edinburgh; http://www.wtcrf.ed.ac.uk) around the ages of ~70 (Wave 1) and ~73 years (Wave 2). It is the Wave 2 data that are examined here. Participants also received detailed brain MRI around the same time (mean interval=65.1 days^16^). Diagnoses of clinical conditions were elicited via interview. Clinical vascular conditions asked about included high blood pressure, diabetes, stroke, high cholesterol and any vascular pathology. All subjects gave written, informed consent.

### Genotyping

Participants were genotyped for *APOE* ɛ on the basis of DNA isolated from whole blood taken from the Wave 1 (age 70) assessment, and genotyped by TaqMan assay (Applied Biosystems, Carlsbad, CA, USA) at the WTCRF genetics core.^15^ *TOMM40* 523 was genotyped by the laboratory of Dr Ornit Chiba-Falek (Duke University, Durham, NC, USA) using a method described previously.^17^

### Cognitive assessment

#### Moray house test no.12

This was completed in June 1947 at a mean age of 11 years. This assessment has a 45-min time limit, has a maximum score of 76 and includes questions with a range of numerical, visuospatial and (primarily) verbal reasoning items.^15^ Scores were adjusted for age in days at the time of assessment, and standardized to an IQ score with a mean of 100 and s.d. of 15 for the whole LBC1936 sample.

#### Assessment of cognitive domains

The tests completed at a mean age of 73 years in Wave 2 are described by Deary *et al.*^14,15^ In brief, the cognitive domains assessed were as follows. Working memory was assessed with digit span backwards and letter-number sequencing (both from the WAIS-III^UK^ battery^18^). Processing speed was assessed using: digit symbol coding and symbol search tests from the WAIS-III^UK^; simple reaction time (RT), and four-choice RT via a self-contained device;^19^ and a visual discrimination task called Inspection Time, which assesses speed of basic visual processing and where higher total scores reflect better performance.^15^ Verbal declarative memory was assessed with the verbal paired associates I and II, and logical memory I and II tests (both from the WMS-III^UK^ battery^20^). Specifically, we summed the respective I/II test scores to create verbal paired associates total scores, and logical memory total scores for each individual. Visuospatial ability and memory were assessed with block design and spatial span (WAIS-III^UK^ and WMS-III^UK^, respectively). Abstract reasoning was assessed using matrix reasoning from the WAIS-III^UK^.

Data reduction was applied to the cognitive test scores using three separate PCAs, which produced the following summary cognitive variables. First, ‘general intelligence' (*g*) included the six nonverbal Wechsler subtests of digit span backwards, matrix reasoning, letter-number sequencing, block design, symbol search and digit symbol coding. Second, ‘processing speed' (*g*_speed_) included symbol search, digit symbol coding, simple RT, four-choice RT and Inspection time.^21^ Third, a ‘memory' factor (*g*_m__emory_) was formed from logical memory, spatial span, verbal paired associates, letter-number sequencing and digit span backwards.^22^ The first unrotated principal component score in each domain accounted for 50–54% of the respective variance, and all individual variables had high loadings on their respective first unrotated components. Strictly speaking, PCA does not produce latent factors, however, we use the term here because it is a common usage.

In each PCA, the *g/g*_speed_/*g*_memory_ scores reflect variance that is shared between their respective lists of included cognitive tests, hence eliminating task-specific variance.^23^ However because different tasks assess specific aspects of mental ability (for example, logical memory explicitly assesses declarative verbal memory), it is still informative to include tests of association between *APOE/TOMM40* genotypes and these individual test-score phenotypes.^24^ Further, the large battery of tests applied to the LBC1936 is rare, and other research teams are likely to test relationships studied here with individual tests. Therefore, to allow comparisons with others' work, we include the individual test results in addition to the component scores.

### Diffusion MRI and tractography

The protocol for DT-MRI processing is described in detail in the MRI protocol paper, including imagine acquisition parameters of the DTI sequence.^16^ Briefly, participants underwent whole-brain diffusion MRI acquired using a GE Signa Horizon HDxt 1.5T clinical scanner (General Electric, Milwaukee, WI, USA). The diffusion MRI data were preprocessed using FSL tools (FMRIB, Oxford, UK; http://www.fmrib.ox.ac.uk). Underlying tractography connectivity data were generated using BedpostX/ProbTrackX with a two-fibre model.^25^ Fourteen tracts were identified using probabilistic neighbourhood tractography, an approach for automatic and reproducible tract segmentation.^26,27^ These tracts were considered to be of relevance to cognitive and brain ageing, on the basis of several lines of evidence.^16^

To make sure that segmented tracts were anatomically plausible representations of the tract-of-interest, a researcher inspected all masks, blind to all other data and excluded tracts with aberrant or truncated pathways. Generally, the probabilistic neighbourhood tractography method, which had been designed specifically for use in older brains, reliably segmented the twelve tracts of interest; tracts that did not meet quality critera (for example, truncation, aberrant route), ranged from 0.3% for the splenium of the corpus callosum, to 16% for the left anterior thalamic radiation (mean=5% (ref. 16)). The protocol paper by Wardlaw *et al.*^16^ is an open access article that details specific methodology of probabilistic neighbourhood tractography for each tract, and displays examples of tracts segmented for use in the LBC1936 sample. Tracts assessed were the genu and splenium of corpus callosum, and bilateral anterior thalamic radiations, ventral and rostral cingulum bundles, arcuate, uncinate and inferior longitudinal fascicule; see Lyall *et al.*^10^ for a detailed examination of associations between *APOE/TOMM40* 523 and average FA scores for these tracts.

### Statistical analysis

#### APOE/TOMM40 analysis

We first tested the effects of *APOE* ɛ4 allele presence vs absence, that is, pooled ɛ2/ɛ4, ɛ3/ɛ4 and ɛ4/ɛ4 genotypes vs pooled ɛ2/ɛ2, ɛ2/ɛ3 and ɛ3/ɛ3.

The variable-length poly-T repeat rs10524523 (‘523') was split into three categories:^28^ ‘Short' (<20T residues; S), ‘Long' (≥20; L) and ‘Very-long' (≥30; VL) of which the S allele may or may not be protective in terms of neurodegenerative pathology.^5^ In the first analytic step applied to the whole sample (‘Step 1'), a general linear model tested for a significant effect of the *TOMM40* 523 genotype (that is, S/S; S/L; L/L; L/VL; VL/VL). To investigate the effects of *TOMM40* 523 repeat length independent of biological variation in *APOE* genotype, analysis then focussed separately on two different *APOE* ɛ genotype subgroups. First, participants with the ɛ3/ɛ4 genotype were analysed (‘Step 2'); the S allele may offset or interact biologically with the ɛ3/ɛ4 ‘risk' genotype.^29^ Finally, analysis focussed on participants with the ‘neutral' *APOE* genotype (ɛ3/ɛ3; ‘Step 3'), because this eliminates variance associated with protective and risk *APOE* alleles.^8^ This subgroup analysis means that any statistically significant effects of *TOMM40* 523 genotype cannot be attributed to variation in *APOE* ɛ status. In steps 2 and 3 (i.e., analysis of *TOMM40* 523 in *APOE* ɛ3/ɛ4 and ɛ3/ɛ3 genotype subgroups, respectively), the L and VL alleles were pooled into an “L*” group, as is relatively common;^10^ participants with the S/S genotype were compared with those carrying only one S allele (pooled S/L and S/VL; hereinafter S/L*), and also against participants carrying no S alleles (pooled L/L, L/VL, and VL/VL; hereinafter L*/L*).^6^ 

#### Covariate models

All covariate models controlled for age and gender (‘Model 1'). Cognitive scores were then corrected for age 11 IQ, to investigate ‘cognitive ageing' (‘Model 2'). Significant associations were then were re-tested controlling for the following covariates in addition to those in Model 2; self-reported history of hypertension, stroke, type 2 diabetes, hypercholesterolaemia and all-inclusive vascular disease (‘Model 3').^30^ This reduces the chance that any significant associations occur as secondary to genetic associations with vascular pathology.

An online calculator was used to perform tests of Hardy–Weinberg equilibrium and determine minor allele frequencies (http://www.had2know.com/academics/hardy-weinberg-equilibrium-calculator-3-alleles.html). Data were otherwise analysed with IBM Statistical Package for the Social Sciences, Version 19.0 (SPSS, Chicago, IL, USA). Specifically, univariate general linear models tested the fixed effects of separate *APOE* and *TOMM40* genotypes upon the outcome variables. Outliers of more than 3.30 s.d. from mean values were removed from all cognitive variables to exclude outlying data; this did not affect any final results. To protect against Type 1 errors, false discovery rate (FDR) was used to estimate the number of truly significant findings in the context of testing multiple associations.^31^ A Microsoft Excel program^32^ was used to conduct classical one-stage FDR based on associations with cognitive abilities. *P*-values <0.05 were considered to be nominally significant. All *P*-values are raw unless stated as being FDR-adjusted.

#### Mediation analysis

**Figure 1:**
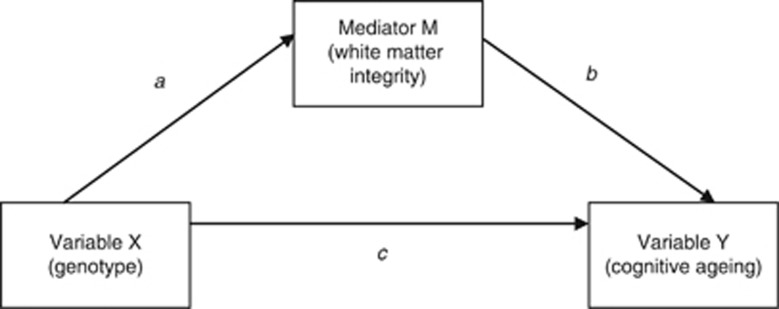
An example mediation model, where variable X's effects (*APOE/TOMM40*) on variable Y (cognitive ageing) can be either direct, or indirect via variable M (white matter integrity). Path *a* represents the effect of X on M, while path *b* represents the effect of M on Y, partialling out the effect of X. The direct effect of X on Y is represented by path *c* (adapted from Preacher and Hayes^33^).

Mediation analysis was used to test the indirect effect of the predictor variable (*APOE/TOMM40*) on the outcome (cognitive ageing), through the hypothesized mediator (white matter integrity). Mediation analysis was run using the INDIRECT bootstrapping macro.^33^ Briefly, variable X's effects (*APOE/TOMM40*) on variable Y (cognitive ageing) can be either direct, or indirect via variable M (white matter integrity). In Figure 1, path *a* represents the effect of X on M, while path *b* represents the effect of M on Y, partialling out the effect of X. The direct effect of X on Y is represented by path *c*. The indirect effect can then be quantified as the combined product of paths *a* and *b*. The bias-corrected bootstrapping point estimate coefficients that are reported here each reflect this indirect product.^33^

Bootstrapping point estimate coefficients were unstandardized and averaged over 5000 bootstrap estimates.^33^ The indirect point estimate coefficients (commonly simply ‘effects') were considered statistically significant if the 95% confidence intervals (CIs) did not cross 0.00.^33^ This method has been used previously to investigate the brain substrates of genetic-cognitive ageing associations.^34^

## Results

### Descriptive statistics

Of the 1091 LBC1936 participants originally enrolled in the study, 866 attended Waves 1 and 2. Individuals who had Mini-Mental State Examination scores <24, a cutoff commonly used to indicate dementia,^35^ did not complete the Mini-Mental State Examination at Wave 2, or had a reported history of dementia were excluded from analysis. Overall, this left 859 participants, of which 811 and 823 participants had successful genotyping for *APOE* and *TOMM40*, respectively.

*APOE* had allele frequencies of ɛ2=7.3%, ɛ3=76.9% and ɛ4=15.8%, with genotype frequencies of: ɛ2/ɛ2=3 (0.4%), ɛ2/ɛ3=95 (11.7%), ɛ2/ɛ4=18 (2.2%), ɛ3/ɛ3=472 (58.2%), ɛ3/ɛ4=208 (25.6%), and ɛ4/ɛ4=15 (1.8%) (total *n*=811). *TOMM40* 523 had allele frequencies of S=41.0%, L=15.4% and VL=43.6%, with genotype frequencies of S/S=125 (15.2%), S/L=123 (14.9%), S/VL=302 (36.7%), L/L=18 (2.2%), L/VL=95 (11.5%) and VL/VL=160 (19.4%) (total *n*=823). Exact tests confirmed that *APOE* and *TOMM40* were in Hardy–Weinberg equilibrium (*P*-values=0.44 and 0.06, respectively).

### APOE, TOMM40 and cognitive ability—not adjusted for childhood intelligence (Model 1)

Significant deleterious/negative effects of the *APOE* ɛ4 allele (vs absence) were found on three tasks: specifically symbol search (*P*=0.048), inspection time (*P*=0.004) and spatial span (*P*=0.033; see Supplementary Table 1). For *TOMM40* 523, no significant effects were found in the whole sample (‘Step 1') or ɛ3/ɛ3 genotype subgroup (‘Step 3'). A single significant protective effect of the S allele was found in *APOE* ɛ3/ɛ4 genotype subgroup only, for letter-number sequencing (*P*=0.035; ‘Step 2' see Supplementary Table 2). Neither *APOE* ɛ nor *TOMM40* 523 genotypes were associated with age 11 IQ scores themselves (*P*>0.05; see Supplementary Tables 1 and 2).

### APOE, TOMM40 and cognitive ability—adjusted for childhood intelligence (Models 2 and 3)

Significant deleterious effects of *APOE* ɛ4 allele presence (vs absence) were found for nine out of a possible fifteen age 73 ‘cognitive ageing' variables (see Table 1). Specifically these were *g* (*P*=0.005), matrix reasoning (*P*=0.041), *g*_speed_(*P*=0.016), digit symbol coding (*P*=0.048), symbol search (*P*=0.014), inspection time (*P*=0.001), *g*_memory_ (*P*=0.020), logical memory Total (*P*=0.013) and spatial span (*P*=0.008; ‘Step 1'). Each significant association survived correction for vascular disease history (*P*<0.05; see Supplementary Table 3).

As shown in Table 2, two significant protective associations with S allele possession were found for *TOMM40* 523: in the whole sample for spatial span (*P*=0.043; ‘Step 1'), and for letter-number sequencing in the *APOE* ɛ3/ɛ4 genotype subgroup only (*P*=0.035; ‘Step 2'). The significant association with spatial span scores did not remain significant when corrected for possession of the *APOE* ɛ4 allele (*P*>0.05). The significant association with letter-number sequencing attenuated to (marginal) nonsignificance when corrected for vascular disease history (*P*=0.050, rounded down). Because of this, associations between *TOMM40* 523 and cognitive ageing were not examined further.

### Correction for multiple testing with FDR

When the above tests of associations were corrected for multiple testing with FDR, all significant associations attenuated to nonsignificance (all FDR-adjusted *P*-values >0.05). Further exploratory analyses were conducted on the basis that they could provide directions for future studies.

### Mediation: intercorrelations between white matter tract integrity and cognitive ageing

Analyses next examined the mediation of genetic-cognitive ageing associations via white matter tract integrity metrics. We examined correlations between white matter tract variables, which showed significant raw deleterious association with *APOE* ɛ4 possession as reported by Lyall *et al.*^10^—namely left inferior longitudinal fasciculus FA and right ventral cingulum FA—and cognitive ageing variables, which were significantly associated with *APOE* ɛ4 in the present report after correction for vascular disease history, that is, those at *P*<0.05 in Table 1 which survived this correction (see Supplementary Table 3 for the exact data).

As can be seen in Table 3, several semi-partial correlations between white matter tract FA metrics and cognitive scores were statistically significant, controlling for age, gender and age 11 IQ, where both the cognitive and imaging variables were also associated with *APOE* ɛ4 (detailed below).

### Mediation: APOE/TOMM40→white matter tract integrity→cognitive ageing

Brain imaging and cognitive variables that showed nominally significant raw associations with *APOE* ɛ4, and that were themselves significantly correlated, were examined further for mediation. Analyses tested exploratory mediation on the basis of the above correlations, with the bootstrapping technique.^33^

**Figure 2:**
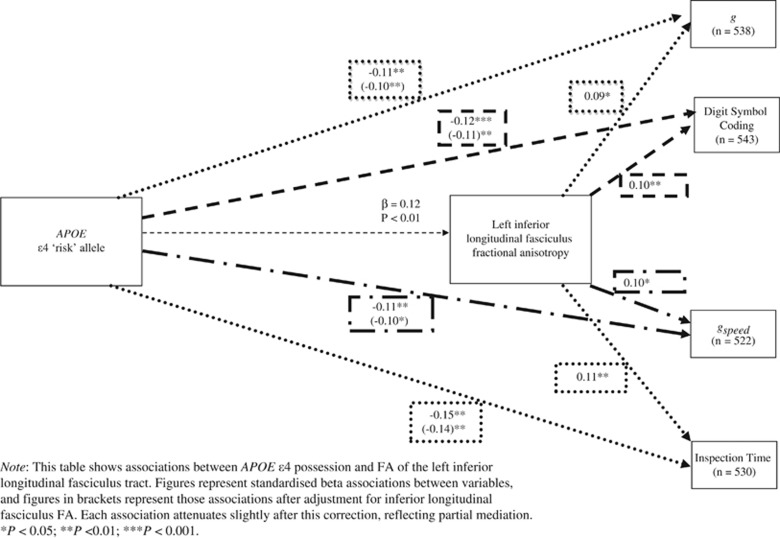
Three-way associations between *APOE* ɛ, left inferior longitudinal fasciculus tract integrity and cognitive ability adjusted for age 11 IQ.

Bootstrapping statistics indicated that left inferior longitudinal fasciculus FA significantly mediated the association between *APOE* and *g* (indirect effect=−0.02, 95% CIs=−0.05 to −0.00), *g*_speed_ (indirect effect=−0.03, 95% CIs=−0.07 to −0.01), digit symbol coding (indirect effect=−0.30, 95% CIs=−0.79 to −0.05) and inspection time (indirect effect=−0.30, 95% CIs=−0.76 to −0.06), but not matrix reasoning (indirect effect=−0.06, 95% CIs=−0.20 to 0.17), symbol search (indirect effect=−0.08, 95% CIs=−0.30 to 0.21), *g*_memory_ (indirect effect=<−0.00, 95% CIs=−0.22 to 0.21) or spatial span (indirect effect=−0.04, 95% CIs=−0.12 to 0.01). Cases of statistically significant mediation are displayed in Figure 2. Bootstrapping statistics indicated that right ventral cingulum FA did not significantly mediate the association between *APOE* and inspection time total scores.

## Discussion

### Overview

This report found significant deleterious effects of *APOE* ɛ4 allele possession on several cognitive tasks, independent of age, gender, childhood IQ and vascular disease history; namely tests of nonverbal reasoning (matrix reasoning), visuospatial ability (spatial span), processing speed (digit symbol coding, symbol search, inspection time), and memory (logical memory total). We also found significant nominal effects of *APOE* genotype on general factors of intelligence, processing speed and memory, constructed with PCA, consistent with recent large studies.^3,36^ The current study did not find significant effects of *TOMM40* 523 genotype (that remained significant when we added the covariates of *APOE* ɛ4 or vascular disease history). Formal tests of mediation with bootstrapping showed that the left inferior longitudinal fasciculus significantly mediated associations between *APOE* ɛ4 and *g*, digit symbol coding, *g*_speed_ and inspection time total scores. This tract did not completely mediate these significant associations; the gene-cognitive associations did not attenuate markedly.

All associations with cognitive ability attenuated to nonsignificance when corrected for type 1 error with FDR. Cognitive variables are strongly intercorrelated, as reflected by the general factors that were constructed with PCA. Although FDR is less affected by test statistics that are based on intercorrelated variables when compared with other type 1 error adjustment procedures such as Bonferroni,^31^ this can still make correction for multiple testing overly conservative.^37^ We interpret all significant raw gene-cognitive associations cautiously, and emphasize that they require independent replications. These intercorrelations also highlight the fact that the genetic associations with cognitive variables are not independent and may partly reflect a degree of shared variance between these tasks (as indicated by *g*).

### Interpretation: APOE ɛ genotype

The demonstration that some significant associations between *APOE* and aspects of ‘cognitive ageing' (cognitive test scores in older age adjusted for childhood intelligence) are mediated by white matter tract integrity is novel, possibly reflecting the fact that relatively few studies have the requisite genetic, imaging and cognitive data.^38^ This is important because it helps to elucidate the neural substrates underpinning the association between *APOE* and cognitive ageing.

It is plausible that the left inferior longitudinal fasciculus at least partly mediates specific *APOE*-cognitive ageing associations. The inferior longitudinal fasciculus is an occipito-temporal tract.^39^ Correlations between white matter integrity and cognitive ability may reflect a ‘computational bottleneck' whereby lowered integrity limits the amount of information that can be transmitted between brain structures (Westlye *et al.,*^40^ pp. 514). This would theoretically strengthen the FA-cognitive correlation:^40^ for example, Ryan *et al.*^13^ reported that associations between FA and cognitive ability in relatively healthy older adults (*N*=126) differed significantly according to *APOE* genotype (for example, frontal lobe FA correlated with an 'Executive function' score at *r=*0.46 for ɛ4 carriers, vs. *r*=0.04 in non-carriers); we indirectly add to this finding with a relatively large study that includes formal tests of mediation.^33^ This theory, overall, provides a reasonable biological explanation for the mediating role of the inferior longitudinal fasciculus and performance on specific cognitive tasks. It is also possible that the integrity of this tract correlates significantly with the actual mechanistic brain phenotype, which was perhaps not examined here.^38^

### Interpretation: TOMM40 ‘523' poly-T repeat genotype

We are aware of three previous independent studies of *TOMM40* 523 genotype and cognitive ability in older adults.^6, 7, 8^ The current findings in the LBC1936 sample contrast with previous studies in showing no significant effects of *TOMM40* 523 genotype, independent of *APOE* or vascular disease history. The relatively small samples reported by Johnson *et al.*^8^ (*N*=117) and Hayden *et al.*^7^ (*N*=127), and the marginal significance reported by Caselli *et al.*^6^ (*P*=0.04) caution that previous significant findings may to an extent reflect type 1 error.

### Limitations and future research

This report examined variables that were statistically significantly intercorrelated at *P*<0.05, and where mediation would therefore be most likely (‘Causal Steps' approach^41^). A limitation of this ‘Causal Steps' approach is that each hypothesis test carries a possibility of type 1 or type 2 error. Rather, Hayes^41^ suggests hypothesis-driven testing of indirect effects, regardless of the statistical significance of the mediator's association with the independent and dependent variables; it is possible that significant *APOE*-cognitive ageing associations occur via very small effects on a large, distributed range of brain phenotypes. Future studies should consider a large-scale examination of *APOE* genotype, different brain imaging phenotypes and cognitive ageing variables, possibly using a structural equation modelling/path analytic framework formally to test the network of associations and for the presence of mediation.

There is evidence that *APOE* genotype may have a significant effect on brain white matter throughout the lifecourse, including early development.^42, 43, 44^ This could imply that ageing effects may be superimposed on underlying vulnerabilities in the way that brain white matter develops in *APOE* ɛ4 carriers (vs non-carriers). The current sample does not have empirical brain imaging at younger ages to address this possibility further.

Although *APOE* ɛ4 is an established risk factor for late onset Alzheimer's disease and for worse cognitive ageing, this polymorphism accounts for only a small fraction of phenotypic variance. Other genetic loci or environmental variables are likely to have significant interactive or independent roles. The approach described here will be useful in testing similar genetic/brain imaging/cognitive associations, however future studies will require large, well-phenotyped cohorts.

## Figures and Tables

**Table 1:** *APOE ɛ* and cognitive ability at age 73 years, adjusted for age 11 IQ

*Cognitive test*	*ɛ4 allele presence (vs absence)*		
	*(d.f.) F statistics*	P	*Partial* η^*2*^
General factor: intelligence (*g*)	**(1, 746)=8.04**	**0.005**	**0.011**
Digit span backwards	(1, 755)=0.53	0.468	0.001
Matrix reasoning	**(1, 754)=4.19**	**0.041**	**0.006**
Block design	(1, 753)=3.70	0.055	0.005
Letter-number sequencing	(1, 754)=0.70	0.403	0.001
General factor: processing speed (*g*_speed_)	**(1, 716)=5.80**	**0.016**	**0.008**
Digit symbol coding	**(1, 753)=3.92**	**0.048**	**0.005**
Symbol search	**(1, 750)=6.04**	**0.014**	**0.008**
Simple reaction time (seconds)	(1, 747)=0.04	0.845	0.000
Four-choice reaction time (seconds)	(1, 751)=0.30	0.587	0.000
Inspection time	**(1, 729)=11.10**	**0.001**	**0.015**
General factor: memory (*g*_m__emory_)	**(1, 739)=5.43**	**0.020**	**0.007**
Logical memory	**(1, 753)=6.23**	**0.013**	**0.008**
Verbal paired associates	(1, 741)=1.03	0.311	0.001
Spatial span	**(1, 751)=7.10**	**0.008**	**0.009**

Age in days at the time of testing, gender and age 11 IQ statistically controlled. Associations significant at *P*<0.05 are in bold.

**Table 2:** *TOMM40 ‘523'* poly-T repeat length genotype and cognitive ability—adjusted for age 11 IQ

*Cognitive test*	*Step 1 Whole sample*			*Step 2 ɛ3/ɛ4 genotype subgroup only*			*Step 3 ɛ3/ɛ3 genotype subgroup only*		
	*(d.f.) F statistics*	P	*Partial* η^*2*^	*(d.f.) F statistics*	P	*Partial* η^*2*^	*(d.f.) F statistics*	P	*Partial* η^*2*^
General factor: intelligence (*g*)	5, 753=1.52	0.182	0.010	(1, 186)=1.04	0.309	0.006	(2, 418)=0.92	0.401	0.004
Digit span backwards	5, 762=0.08	0.995	0.001	(1, 187)=0.07	0.786	0.000	(2, 426)=0.74	0.476	0.003
Matrix reasoning	5, 761=0.73	0.602	0.005	(1, 187)=1.02	0.314	0.005	(2, 425)=0.56	0.572	0.003
Block design	5, 760=1.39	0.228	0.009	(1, 187)=0.25	0.618	0.001	(2, 424)=1.31	0.277	0.006
Letter-number sequencing	5, 761=1.19	0.312	0.008	**(1, 187)=4.51**	**0.035**	**0.024**	(2, 425)=1.39	0.250	0.007
General factor: processing speed (*g*_s__peed_)	5, 723=0.93	0.463	0.006	(1, 173)=0.31	0.578	0.002	(2, 407)=0.30	0.739	0.001
Digit symbol coding	5, 760=0.93	0.459	0.006	(1, 187)=0.31	0.578	0.002	(2, 424)=0.89	0.410	0.004
Symbol search	5, 757=1.18	0.316	0.008	(1, 186)=0.00	0.951	0.000	(2, 422)=0.37	0.692	0.002
Simple reaction time (seconds)	5, 754=0.55	0.741	0.004	(1, 185)=0.42	0.518	0.002	(2, 421)=1.25	0.287	0.006
Four-choice reaction time (seconds)	5, 758=0.35	0.880	0.002	(1, 186)=0.84	0.462	0.004	(2, 424)=0.44	0.646	0.002
Inspection time	5, 736=2.13	0.060	0.014	(1, 177)=2.26	0.135	0.013	(2, 414)=0.20	0.820	0.001
General factor: memory (*g*_memory_)	5, 746=2.11	0.063	0.014	(1, 183)=0.17	0.685	0.001	(2, 416)=0.79	0.457	0.004
Logical memory	5, 760=2.13	0.060	0.014	(1, 186)=0.27	0.601	0.001	(2, 425)=2.33	0.099	0.011
Verbal paired associates	5, 748=0.95	0.449	0.006	(1, 184)=0.02	0.891	0.000	(2, 417)=1.09	0.337	0.005
Spatial span	**5, 758=2.31**	**0.043**	**0.015**	(1, 187)=3.09	0.081	0.016	(2, 422)=1.25	0.288	0.006

Age in days at the time of testing, gender and age 11 IQ statistically controlled. Associations significant at *P*<0.05 are in bold.

**Table 3:** Intercorrelations between cognitive ageing and white matter tract variables that are each significantly associated with *APOE* ɛ genotype

r *(P-value)*	*Right ventral cingulum FA*	*Left inferior longitudinal fasciculus FA*
General factor: intelligence (*g*)	0.02 (0.347)	**0.20 (<0.001)**
Matrix reasoning	−0.01 (0.411)	**0.10 (0.008)**
General factor: processing speed (*g*_s__peed_)	0.04 (0.196)	**0.17 (<0.001)**
Digit symbol coding	0.03 (0.276)	**0.18 (<0.001)**
Symbol search	−0.03 (0.240)	**0.11 (0.004)**
Inspection time	**0.07 (0.042)**	**0.14 (<0.001)**
General factor: memory (*g*_m__emory_)	0.01 (0.441)	**0.08 (0.024)**
Logical memory	0.02 (0.328)	0.05 (0.131)
Spatial span	−0.02 (0.334)	**0.09 (0.015)**

Abbreviation: FA, fractional anisotropy.

Pearson semi-partial correlations controlling for age, gender and age 11 IQ. Figures reflect ‘*r*' correlations. FA, fractional anisotropy. Significant correlations in bold reflect a significant correlation (*P*<0.05) where both variables are also associated with *APOE* ɛ4 independent of vascular disease history (Supplementary Table 3).

## Notes

ADR is the CEO and only stock holder of Zinfandel Pharmaceuticals, a company in an Alliance with Takeda Pharmaceuticals, to perform the prospective qualification of the *TOMM40* marker for age of onset distribution of Alzheimer's disease. For this study, Zinfandel Pharmaceuticals paid for the *TOMM40* assays to be performed for medical research, not as a clinical diagnostic. AMS is the spouse of ADR, and AMS and MWL are consultants to Zinfandel Pharmaceuticals. The remaining authors declare no conflict of interest.
