# Supplementary material for: Are APOE ɛ genotype and TOMM40 poly-T repeat length associations with cognitive ageing mediated by brain white matter tract integrity?
Source: Transl Psychiatry. 2014 Sep 23;4(9):e449–. doi: 10.1038/tp.2014.89 (PMC4203017; doi:10.1038/tp.2014.89)
Supplement: Supplementary Tables 1-3 [file tp201489x1.doc]

**Supplementary Table 1.** *APOE ε* and cognitive ability at age 73 years - not adjusted for age 11 IQ.

| Cognitive test | ε4 allele presence (vs. absence) | | |
| --- | --- | --- | --- |
|  | (d.f.) F statistics | *P* | Partial η^2^ |
| Age 11 IQ | (1, 757) = 0.75 | 0.386 | 0.001 |
| General factor: intelligence (g) | (1, 797) = 0.19 | 0.166 | 0.002 |
| Digit Span Backwards | (1, 807) = 0.03 | 0.862 | 0.000 |
| Matrix Reasoning | (1, 806) = 1.40 | 0.237 | 0.002 |
| Block Design | (1, 805) = 1.77 | 0.184 | 0.002 |
| Letter-Number Sequencing | (1, 806) = 0.10 | 0.747 | 0.000 |
| General factor: processing speed (g_Speed_) | (1, 765) = 2.53 | 0.112 | 0.003 |
| Digit Symbol Coding | (1, 804) = 0.67 | 0.414 | 0.001 |
| Symbol Search | **(1, 802) = 3.92** | **0.048** | **0.005** |
| Simple Reaction time (seconds) | (1, 798) = 0.01 | 0.914 | 0.000 |
| Four Choice Reaction Time (seconds) | (1, 802) = 0.10 | 0.748 | 0.000 |
| Inspection Time | **(1, 799) = 8.42** | **0.004** | **0.011** |
| General factor: memory (g_Memory_) | (1, 788) = 1.94 | 0.165 | 0.002 |
| Logical Memory | (1, 805) = 0.32 | 0.075 | 0.004 |
| Verbal Paired Associates | (1, 790) = 0.27 | 0.603 | 0.000 |
| Spatial Span | **(1, 803) = 0.46** | **0.033** | **0.006** |

*Note*. Age in days at time of testing and gender statistically controlled. Associations significant at *P* < 0.05 are printed in bold-face and italics.

**Supplementary Table 2** *TOMM40 ‘523’* poly-T repeat length genotype and cognitive ability - not adjusted for age 11 IQ.

| Cognitive test | Step 1  Whole sample | | | Step 2  ε3/ε4 genotype subgroup only | | | Step 3  ε3/ε3 genotype subgroup only | | |
| --- | --- | --- | --- | --- | --- | --- | --- | --- | --- |
|  | (d.f.) F statistics | *P* | Partial η^2^ | (d.f.) F statistics | *P* | Partial η^2^ | (d.f.) F statistics | *P* | Partial η^2^ |
| Age 11 IQ | 5, 763 = 0.31 | 0.908 | 0.002 | (1, 188) = 0.00 | 0.985 | 0.000 | (2, 427) = 0.88 | 0.417 | 0.004 |
| General factor: intelligence (g) | 5, 805 = 0.49 | 0.784 | 0.003 | (1, 198) = 0.70 | 0.405 | 0.004 | (2, 449) = 0.72 | 0.489 | 0.003 |
| Digit Span Backwards | 5, 815 = 0.10 | 0.992 | 0.001 | (1, 200) = 0.07 | 0.788 | 0.000 | (2, 458) = 0.53 | 0.591 | 0.002 |
| Matrix Reasoning | 5, 814 = 0.59 | 0.707 | 0.004 | (1, 200) = 1.65 | 0.201 | 0.008 | (2, 457) = 0.43 | 0.654 | 0.002 |
| Block Design | 5, 813 = 0.74 | 0.597 | 0.005 | (1, 200) = 0.35 | 0.556 | 0.002 | (2, 455) = 1.20 | 0.301 | 0.005 |
| Letter-Number Sequencing | 5, 814 = 1.07 | 0.378 | 0.007 | **(1, 200) = 4.48** | **0.035** | **0.022** | (2, 457) = 1.28 | 0.278 | 0.006 |
| General factor: processing speed (g_Speed_) | 5, 773 = 0.42 | 0.839 | 0.003 | (1, 185) = 0.16 | 0.691 | 0.001 | (2, 437) = 0.06 | 0.944 | 0.000 |
| Digit Symbol Coding | 5, 812 = 0.73 | 0.603 | 0.004 | (1, 199) = 0.32 | 0.570 | 0.002 | (2, 456) = 0.99 | 0.371 | 0.004 |
| Symbol Search | 5, 810 = 0.83 | 0.526 | 0.005 | (1, 199) = 0.01 | 0.916 | 0.000 | (2, 454) = 0.59 | 0.554 | 0.003 |
| Simple Reaction time (seconds) | 5, 806 = 0.45 | 0.816 | 0.003 | (1, 198) = 0.28 | 0.596 | 0.001 | (2, 452) = 0.97 | 0.382 | 0.004 |
| Four Choice Reaction Time (seconds) | 5, 810 = 0.38 | 0.860 | 0.002 | (1, 198) = 0.95 | 0.332 | 0.005 | (2, 456) = 0.25 | 0.783 | 0.001 |
| Inspection Time | 5, 787 = 1.79 | 0.113 | 0.011 | (1, 189) = 1.57 | 0.211 | 0.008 | (2, 445) = 0.36 | 0.700 | 0.002 |
| General factor: memory (g_Memory_) | 5, 796 = 1.27 | 0.276 | 0.008 | (1, 195) = 0.16 | 0.691 | 0.001 | (2, 446) = 0.43 | 0.650 | 0.002 |
| Logical Memory | 5, 813 = 1.50 | 0.189 | 0.009 | (1, 199) = 0.09 | 0.767 | 0.000 | (2, 457) = 0.53 | 0.587 | 0.002 |
| Verbal Paired Associates | 5, 798 = 0.68 | 0.640 | 0.004 | (1, 196) = 0.02 | 0.899 | 0.000 | (2, 447) = 1.01 | 0.365 | 0.004 |
| Spatial Span | 5, 811 = 2.21 | 0.051 | 0.004 | (1, 200) = 3.48 | 0.064 | 0.017 | (2, 454) = 0.85 | 0.428 | 0.004 |

*Note*. Age in days at time of testing and gender statistically controlled. Associations significant at *P* < 0.05 are printed in bold-face.

**Supplementary Table 3** Significant associations between *APOE* and cognitive ageing in Table 1 – adjusted for vascular disease history (in addition to age, gender, and age 11 intelligence).

| *Cognitive ageing*  *variable* | *APOE* ε  ε4+ vs. ε4- | | |  |
| --- | --- | --- | --- | --- |
|  | (d.f.) F statistics | *P* | Partial η^2^ |  |
| General factor: intelligence (g) | 1, 741 = 8.47 | 0.004 | 0.011 |  |
| Matrix Reasoning | 1, 749 = 4.41 | 0.036 | 0.006 |  |
| General factor: processing speed (g_Speed_) | 1, 711 = 6.36 | 0.012 | 0.009 |  |
| Digit Symbol Coding | 1, 748 = 4.27 | 0.039 | 0.006 |  |
| Symbol Search | 1, 745 = 5.72 | 0.017 | 0.008 |  |
| Inspection Time | 1, 724 = 11.36 | 0.001 | 0.015 |  |
| General factor: memory (g_Memory_) | 1, 745 = 5.36 | 0.021 | 0.007 |  |
| Logical Memory | 1, 748 = 6.22 | 0.013 | 0.008 |  |
| Spatial Span | 1, 746 = 6.03 | 0.014 | 0.008 |  |
